# Effect of dexamethasone on antibody response of horses to vaccination with a combined equine influenza virus and equine herpesvirus‐1 vaccine

**DOI:** 10.1111/jvim.16978

**Published:** 2023-12-23

**Authors:** Nicole Kreutzfeldt, Thomas M. Chambers, Stephanie Reedy, Kennedy Michelle Spann, Nicola Pusterla

**Affiliations:** ^1^ William R. Pritchard Veterinary Medical Teaching Hospital, School of Veterinary Medicine University of California Davis California USA; ^2^ Maxwell H. Gluck Equine Research Center University of Kentucky Lexington Kentucky USA; ^3^ School of Veterinary Medicine University of California Davis California USA; ^4^ Department of Medicine and Epidemiology, School of Veterinary Medicine University of California Davis California USA

**Keywords:** corticosteroids, horse, immune, medicine

## Abstract

**Background:**

Dexamethasone is routinely administered to horses but its effect on the antibody response to a commercial EIV/EHV vaccine is unclear.

**Hypothesis:**

Horses receiving dexamethasone will have lower postvaccination antibody levels against EIV and EHV‐1 than vaccinated controls.

**Animals:**

Fifty‐five healthy adult research horses.

**Methods:**

Randomized cohort study. Control (no vaccine, group 1), vaccination only (EIV/EHV‐1/EHV‐4, Prestige 2, Merck Animal Health, group 2), vaccination and concurrent single intravenous dose of dexamethasone (approximately .05 mg/kg, group 3), vaccination and 3 intravenous doses of dexamethasone at 24 hours intervals (group 4). Serum SAA levels were measured on day 1 and day 3. Antibody levels against EIV (hemagglutination inhibition assay, Kentucky 2014 antigen) and EHV‐1 (multiplex ELISA targeting total IgG and IgG 4/7) were measured on day 1 and day 30.

**Results:**

Significantly increased mean antibody titers after vaccination were only noted against EIV and only after the vaccination alone (n = 14, prevaccine mean [prvm] 166.9, SD 259.6, 95% CI 16.95‐316.8; postvaccine mean [povm] 249.1, SD 257.2, 95% confidence interval [CI] 100.6‐397.6, *P* = .02) and the single dose dexamethasone (n = 14, prvm 93.14, SD 72.2, CI 51.45‐134.8; povm 185.1, SD 118, CI 116.7‐253.6, *P* = .01), but not after multiple doses of dexamethasone (n = 14, prvm 194.3, SD 258.3, CI 45.16‐343.4; povm 240.0, SD 235.7, CI 103.9‐376.1, *P* > .05).

**Conclusion:**

The effect of dexamethasone on the postvaccine antibody response varies depending on the dosing frequency and the antigen‐specific antibody type.

**Abbreviations**
CD4: cluster of differentiation 4
EHV: equine herpes virus
EIV: equine influenza virus
ELISA: enzyme‐linked immunosorbent assay
IgG: immunoglobulin G
PRE: pura raza Espanola
SAA: serum amyloid A

## 1 INTRODUCTION

Glucocorticosteroids, such as dexamethasone, are frequently used in equine medicine and have long been recognized for their anti‐inflammatory and immunosuppressive properties to treat a variety of clinical conditions, including asthma, allergic reactions, inflammatory bowel disease or neoplastic diseases such as lymphoma. Knowledge about the effect of corticosteroids, particularly dexamethasone, on a horse's antibody response to vaccination is currently limited. Inhalant fluticasone appears to have no effect on the humoral or cell‐mediated response to vaccines in horses.^1^ Administration of a single dose of dexamethasone is associated with decreased CD4+ T‐cell production and reduced levels of inflammatory cytokines in a healthy horse.^2^ There are lower levels of specific IgG subtypes in response to different bovine vaccines when horses are repeatedly administered dexamethasone intramuscularly over a period of 8 weeks following vaccination,^3^ although the overall serum titers of IgG are similar to the vaccinated control group.

In human medicine, the role of immunosuppressants administered around the time of vaccination is likewise unclear. Several studies that investigated the immunosuppressive effects of corticosteroids administered orally or by inhalation on the response to different vaccinations in people, mostly inactivated influenza and COVID vaccines, fail to show a consistently diminished immune response in treated patients.^4^, ^5^, ^6^, ^7^, ^8^, ^9^ The Centers for Disease Control and Prevention (CDC) state that there are currently no specific recommendations regarding the use of corticosteroids before or after vaccinations, although suggestions are made to reduce the use of corticosteroids in these situations whenever possible.^10^

Equine influenza and equine herpesvirus‐1 infection are endemic and widespread viral diseases of equids. Outbreaks of disease can result in life‐threatening disease (EHV‐associated myeloencephalopathy, Sunshine tour in Valencia, Spain, February 2021^11^) or substantial economic loss through the interruption of horse trade and movement and the canceling of events during the EIV outbreak in NSW, Australia in 2007/08^12^ or the EIV outbreak in the United Kingdom and Europe in 2018/19.^13^, ^14^, ^15^ An adequate immune response to commercially available vaccines is vital to prevent disease in individual horses and to ensure the health of the general horse population. Investigating the effects of clinically relevant doses of dexamethasone on the antibody response to commonly used vaccines for EIV and EHV‐1/−4 will help guide the recommendations for treatment with dexamethasone around the time of vaccination in horses.

We hypothesized that the intravenous administration of dexamethasone at the time of vaccination would result in lower antibody titers 30 days after vaccination when compared to horses that only received the vaccine. We further hypothesized that the intravenous administration of dexamethasone at the time of vaccination would result in a reduced systemic inflammatory response characterized by lower SAA measurements when compared to horses that received only the vaccine.

## 2 MATERIALS AND METHODS

### 2.1 Animals

Fifty‐five healthy adult horses from the research herd of the Center for Equine Health (CEH) of the School of Veterinary Medicine of California, Davis, were enrolled in the randomized cohort study. This was a convenience sample based on the availability of horses and the results of a power calculation using Mead's resource equation, suggesting that a sample size of 60 horses (15 horses per group) would result in a power of 0.8 with significance set at *P* < 0.05. The study population included 26 geldings and 29 mares, and the represented breeds were 20 Thoroughbreds, 14 Warmbloods, 12 Quarter horses, 4 Standardbreds, and 1 Arabian, 1 American Paint, 1 PRE, 1 Appaloosa and 1 Percheron. The median age of all study horses and within each study group was 14 years (6 years to 21 years; Supplementary Information 1). All horses had received a primary course of vaccination against equine influenza virus (EIV) and equine herpes viruses (EHV‐1/4) in the past consisting of at least 3 doses of a commercial vaccine against EIV/EHV‐1/EHV‐4 (Fluvac Innovator EHV‐4/1, Zoetis^16^), and the last vaccine booster had been administered 6 months before the study begin.

### 2.2 Study design

All horses enrolled in the study were deemed clinically healthy based on clinical examination on the first day of the study and review of recent clinical history. The horses were then randomly assigned to 1 of 4 study groups. Groups were matched by median age as well as sex and breed distribution. Group 1 served as control and did not receive any medication or vaccine during the study period. Horses in group 2 were administered an intramuscular commercial EIV/EHV‐1/EHV‐4 vaccine (Prestige 2, Merck Animal Health Intervet Inc, Madison, New Jersey, https://merckusa.cvpservice.com) on day 1, horses in group 3 received both the vaccine and a single intravenous injection of dexamethasone (Dexamethasone Injection, Sparhawk Laboratories, Inc, Lenexa, Kansas, 2 mg/mL, 20 mg per horse, approximately .05 mg/kg per horse and dose) at the same time on day 1. Horses in group 4 received both the vaccine and an intravenous injection of dexamethasone (as described above) at the same time on day 1, as well as 2 further injections of dexamethasone every 24 hours on day 2 and day 3, for a total of 3 doses of dexamethasone. A dose of .05 mg/kg dexamethasone is commonly used to treat inflammatory conditions in horses.^17^, ^18^ During the study period, horses were housed in either individual outdoor pens or group paddocks or kept on group pasture. Horses did not leave the property and new horses were not introduced to the paddock or pasture groups for the duration of the study. The diet consisted of grass for pasture horses and grass hay and alfalfa hay for horses kept in pens or group paddocks.

### 2.3 Sample collection

Sample collection for all horses was performed during the month of November of the same year. Whole blood samples were collected from each horse on day 1 of the study before any vaccines or dexamethasone were administered for measurement of SAA (Stablelab, Zoetis, Inc, Kalamazoo, Michigan^19^) and antibodies against EIV (hemagglutination inhibition assay, Maxwell H. Gluck Equine Research Center, Kentucky, United States, using Influenza A/equine/Kentucky/2014 [H3N8] as virus antigen^20^) and EHV‐1 (multiplex ELISA targeting total IgG and IgG 4/7 against EHV‐1, University of Cornell, Ithaca, United States,^21^). For the hemagglutination assay, serum was pretreated with potassium periodate to remove nonspecific inhibitors of hemagglutination.^22^ Investigators conducting the HI assays were blinded as to the horse groups and time of sample collection. Whole blood samples were collected again from all study horses for repeat SAA measurements at 72 hours. This time point was chosen based on the results of a previous study that noted peak concentration of serum SAA around 72 hours following vaccination in horses.^23^ On day 30, additional blood samples were obtained from all study horses for repeat measurements of antibody titers against EIV and EHV‐1. The blood serum samples for antibody measurements on day 1 were centrifuged upon collection, the serum was separated into 3 aliquots per sample and subsequently frozen for 30 days at −80°C, to be then submitted with the serum samples obtained on day 30 for paired analysis. The samples for SAA measurements were processed on the day of collection. Following the vaccination, horses were monitored daily for clinical signs of vaccine reactions such as swelling or pain at the injection site, lethargy or fever for 3 days. Additionally, all horses were monitored daily throughout the study period for clinical signs of respiratory infections such as nasal discharge or coughing or evidence of laminitis such as lameness or reluctance to move. The study design was approved by the Institutional Animal Care and Use Committee (IACUC) of the University of California, Davis.

### 2.4 Statistical analysis

All data were analyzed using commercially available statistical software (Prism by GraphPad, version 9.4.1). Descriptive statistics were used to summarize categorical data such as breed, sex and age and this data was presented as absolute value (n) and percentage. Data was assessed for normality using the Shapiro‐Wilk test. Not normally distributed data was analyzed using the Kruskal‐Wallis test to determine significant differences between the antibody titers and SAA levels, respectively, pre‐ and postvaccination in the individual study groups. A 2‐way ANOVA test was used to compare the antibody titers and SAA measurements among the different study groups at the different time points. A value of *P* < .05 was considered significant.

## 3 RESULTS

**FIGURE 1:**
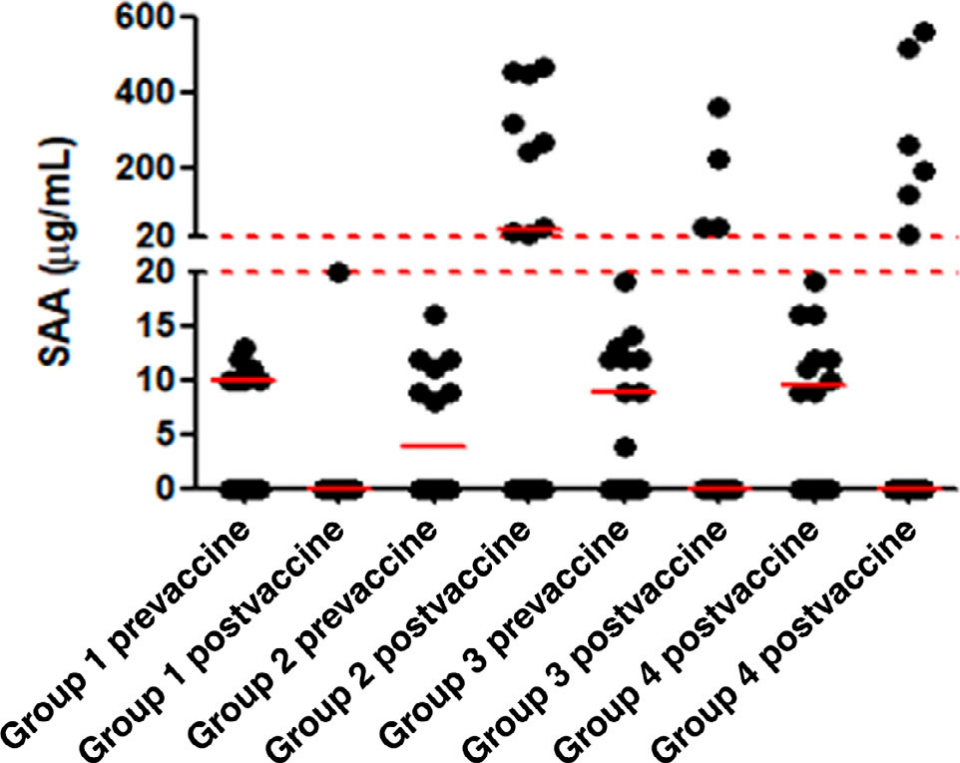
SAA measured pre‐ and 72 hours after vaccine and dexamethasone administration in 55 horses. Horses in group 1 were unvaccinated controls, horses of group 2 received the Prestige 2 EHV 1/EHV 4/EIV vaccine, horses of group 3 received the Prestige 2 vaccine and a single dose of dexamethasone and group 4 horses received the Prestige 2 vaccine and 3 daily intravenous injections of dexamethasone. The solid horizontal red lines represent the median values for each group. The dashed lines indicate a change in scale of the figure to accommodate the higher SAA values.

All 55 horses completed the study and did not show any clinical adverse reactions to the vaccine or the administration of dexamethasone. Baseline serum SAA levels measured before the vaccination were within normal limits and not statistically different among the 4 study groups (*P* > .05; Figure 1). Only horses in group 2 showed a significant increase in serum SAA levels measured at 72 hours after vaccination (*P* = .04). The SAA levels of horses in group 3 and 4 that received dexamethasone at the time of vaccination were not significantly different from those of the control horses at 72 hours (*P* > .05). There was no statistically significant difference (*P* > .05) between the serum SAA levels between group 3 (single dose of dexamethasone) and group 4 (multiple doses of dexamethasone).

**FIGURE 2:**
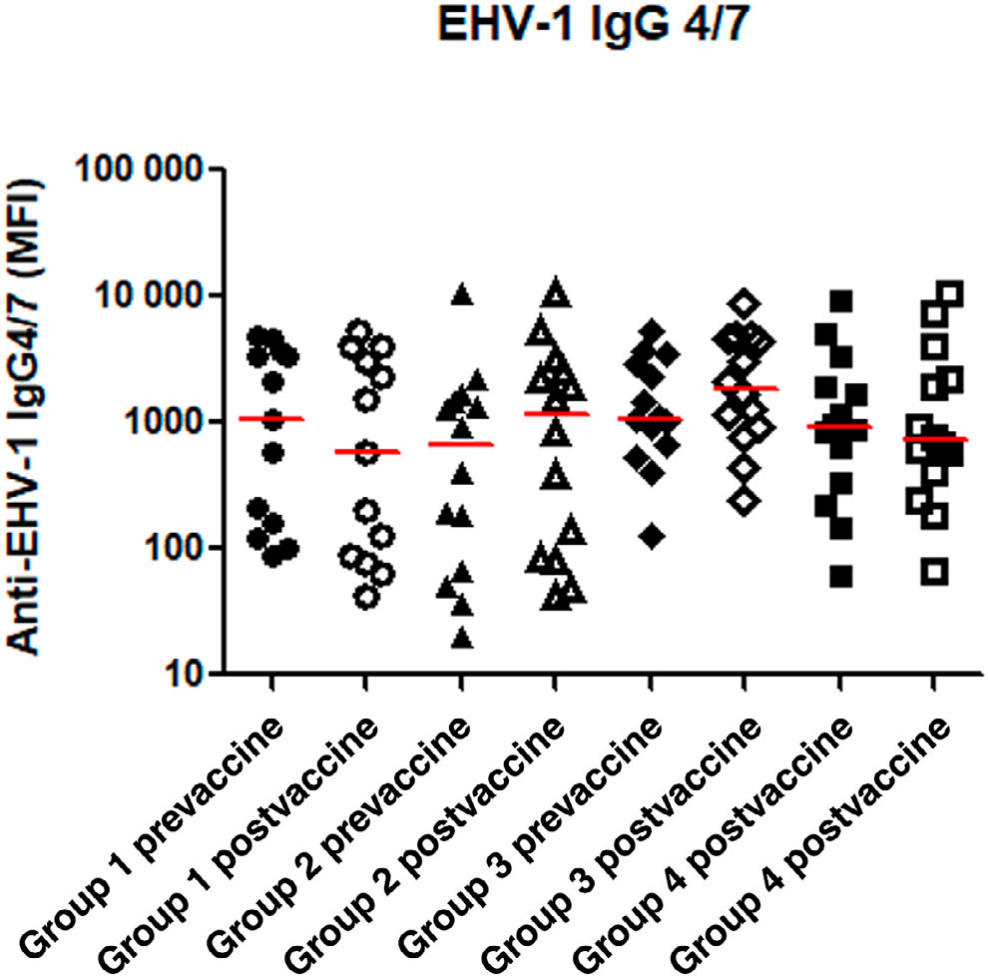
Serum Ig 4/7 measured by EHV‐1 multiplex assays in sentinel horses (group 1, circles), horses vaccinated with Prestige 2 (group 2, triangles), vaccinated horses receiving a single intravenous injection of dexamethasone (group 3, diamonds) and vaccinated horses receiving multiple intravenous injections of dexamethasone (group 4, squares). Pre represents day 0 (before vaccine administration, solid) and post represents 30‐days postvaccine administration (open). The results are expressed as median fluorescence intensity (MFI). The horizontal red bars represent median MFI for each group.

**FIGURE 3:**
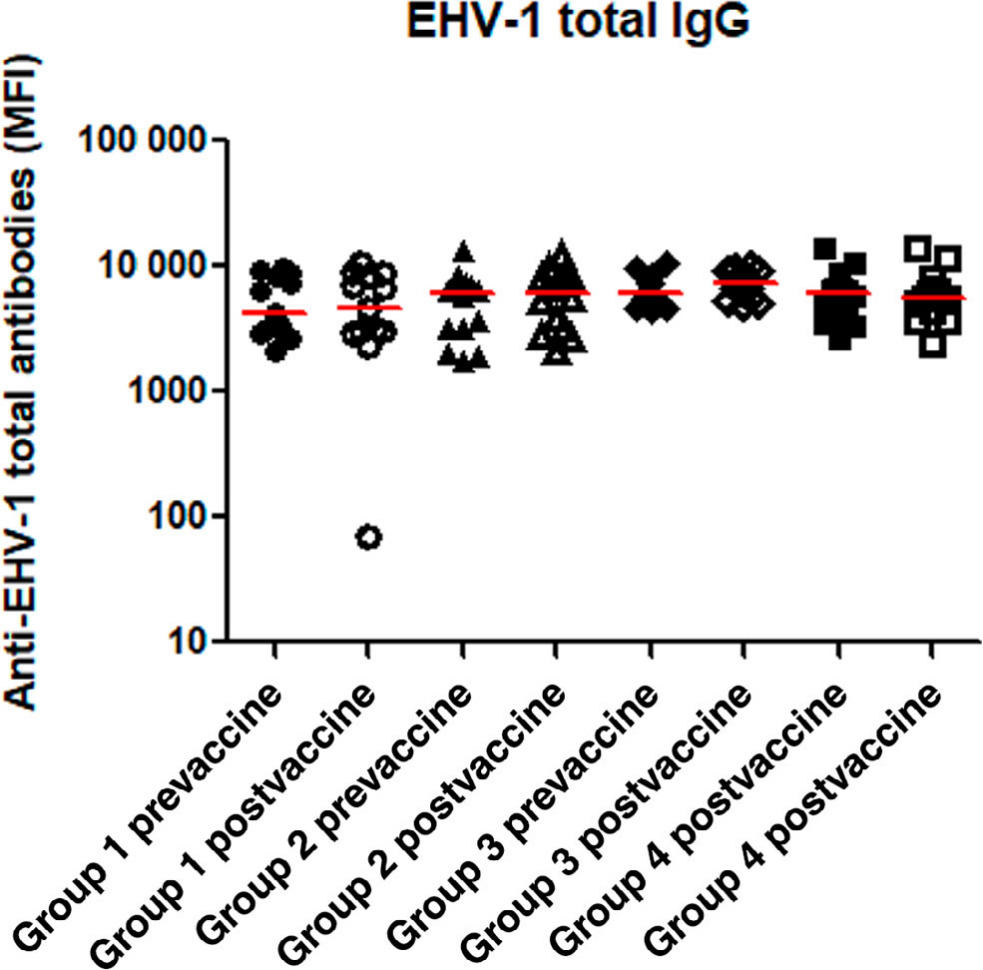
Total serum Ig measured by EHV‐1 multiplex assays in sentinel horses (group 1, circles), horses vaccinated with Prestige 2 (group 2, triangles), vaccinated horses receiving a single intravenous injection of dexamethasone (group 3, diamonds) and vaccinated horses receiving multiple intravenous injections of dexamethasone (group 4, squares). Pre represents day 0 (before vaccine administration, solid) and post represents 30‐days postvaccine administration (open). The results are expressed as median fluorescence intensity (MFI). The horizontal red bars represent median MFI for each group.

**FIGURE 4:**
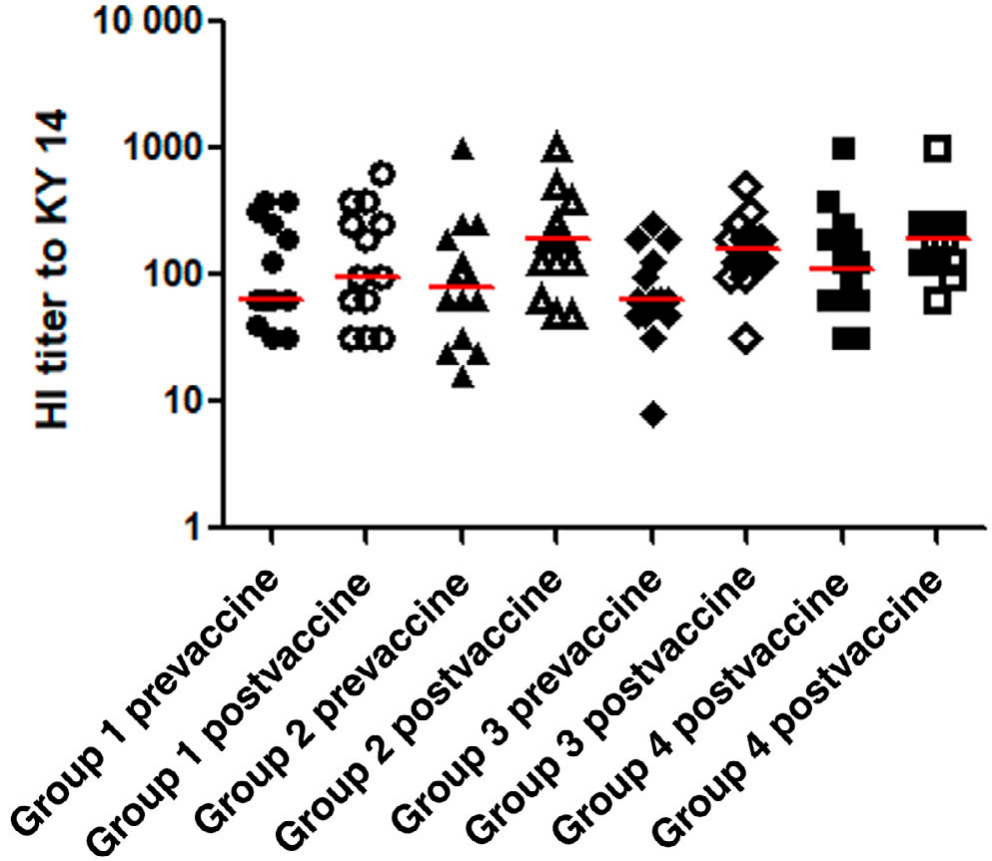
Pre‐ and postvaccine antibody titers against KY/14 in 55 horses. Horses in group 1 (circles) were unvaccinated controls, horses of group 2 (triangles) received Prestige 2 vaccine, horses of group 3 (diamonds) received Prestige 2 vaccine and a single dose of dexamethasone and group 4 horses (squares) received Prestige 2 vaccine and 3 daily intravenous injections of dexamethasone. Pre represents day 0 (before vaccine administration, solid) and post represents 30‐days postvaccine administration (open). The horizontal red bars represent median HI titers for each group.

Baseline serum IgG antibody titers against EHV‐1 total IgG and IgG 4/7 and EIV antibodies measured at day 1 were not significantly different among the 4 study groups (*P* > .05). At day 30 of the study period, the horses in all 4 groups did not have a statistically significant increase in serum titers against‐EHV‐1 (IgG and IgG 4/7; *P* > .05; Figures 2 and 3) compared to baseline (day 1). Among the vaccinated horses, the total IgG and IgG 4/7 titers against EHV‐1 were not significantly different at day 30 between the 2 groups that received dexamethasone and the horses in group 2 that did not receive dexamethasone (*P* > .05). The postvaccination antibody titers against EIV were significantly increased from prevaccination levels in the vaccinated horses of both group 2 (vaccination only; *P* = .02) and group 3 (single dose of dexamethasone, *P* = .01), but not in the vaccinated horses receiving multiple doses of dexamethasone (group 4) and in the sentinel group 1 (Figure 4; Supplementary Tables 1 and 2).

## 4 DISCUSSION

Based on the current study, dexamethasone appeared to decrease the postvaccine inflammatory response in the study horses. Serum SAA levels were only significantly increased in vaccinated horses that were not treated with dexamethasone. Vaccinations induce an acute phase response in horses and result in significantly increased serum SAA levels up to several days after vaccination.^23^, ^24^, ^25^ The half‐life of dexamethasone in horses after a single intravenous injection ranges from 18 minutes to 5.1 hours,^26^ although the duration of any anti‐inflammatory effects after a single administration is difficult to measure. Based on the prolonged suppression of endogenous hydrocortisone^26^ and altered concentrations of cortisol and selected inflammatory and immunomodulatory cytokine after a single intravenous dose of dexamethasone in horses,^27^ a measurable anti‐inflammatory effect can persist for up to 72 to 96 hours. This likely explains the lack of an increase in SAA levels 72 hours postvaccination in the treated horses in this study.

In contrast to the clear effect of dexamethasone on the SAA concentrations in this study, the effect of dexamethasone on the antibody response to vaccination appeared more variable. The postvaccination titers of the 2 measured antigen‐specific antibodies (EHV‐1 and EIV) did not show the same changes throughout the study group in response to the administration of dexamethasone. Antibody levels against EHV were not significantly different after vaccination, whether or not the horses received dexamethasone. However, the postvaccination antibody titers against EIV differed depending on whether the horse's received dexamethasone or not. Additionally, differences in postvaccination antibody titers against EIV were noted with different dosing frequency of dexamethasone (single versus multiple doses). The horses receiving multiple doses of dexamethasone did not develop significantly increased titers against EIV after vaccination, but the horses treated with a single dose, or no dexamethasone did. These observations suggest that any potential modulation of the equine antibody response by dexamethasone is likely complex, and, among others, could be influenced by the antibody subtype and the dosing frequency.

Vaccines using in‐activated, nonreplicating virus such as the study vaccine can stimulate a strong humoral immune response by activating B‐cells and trigger the production of antigen‐specific antibodies.^28^, ^29^ While live vaccines can trigger an immune response through damage recognition patterns associated with the viral antigen, killed and inactivated vaccines commonly rely on adjuvants to increase the efficacy of vaccines. A variety of adjuvants are used in equine vaccinations to potentiate the local inflammatory response and cytokine release, thereby attracting more antigen presenting cells (APC) and resulting in increased antigen uptake, presentation and subsequent B‐cell recruitment and stimulation.^30^, ^31^, ^32^ Activated B‐cells will either immediately produce first‐line antibodies, mostly of the IgM subtype, as an immediate response, or develop a germinal center, undergo somatic hypermutation, maturation and switching of isotypes over several days after antigen stimulation. The latter process is not immediate but results in the production of antigen specific antibodies with high affinity and avidity, as well as memory cell formation.^33^ A local inflammatory response is therefore considered vital for a successful humoral immune response. This suggests that dexamethasone could reduce the immune response in horses both by decreasing the local inflammation incited by the vaccine and through its interference with the signaling cascade and antigen processing.

In the current study, we used an inactivated EIV/EHV‐1/EHV‐4 vaccine and did not observe a lower antibody response against EHV in the study horses treated with dexamethasone. Although a molecular analysis of immune cell subtypes and immune cell function was not performed in this study, these results indicate that dexamethasone did not interfere with the immune response against EHV‐antigen in these horses. These results are in line with previous studies in horses that did not note any decrease in overall antibody titers after treatment with dexamethasone^1^, ^3^ and current recommendations in human medicine published by the CDC (Centers for Disease Control and Prevention), that state that treatment with corticosteroids can be continued when administering inactivated vaccines to people.^34^ While the absence of detectable changes in antibody levels against EHV could indicate the lack of a measurable effect of dexamethasone on the immune response, it could also be related to the dose used in the current study. A dose‐dependent immune‐modulatory effect of dexamethasone has been postulated, although information is limited. A dose‐dependent decrease in T‐cell and B‐cell subsets is noted after a single intravenous dose of dexamethasone administered to healthy human volunteers (2‐8 mg per adult, approximately 0.02‐0.1 mg/kg based on the average body weight of participants), with higher doses resulting in lower cell counts.^35^ The dose of 0.05 mg/kg of dexamethasone used in this study is used in equine medicine for the treatment of various inflammatory conditions including skin and airway inflammation^17^, ^18^ but higher doses up to 0.2 mg/kg are described in this review and could potentially have a different effect on the equine immune response. However, antibody titers against EHV were not only unchanged in the treated horses, but also in the control horses that received only the vaccination and therefore would have been suspected to produce increased antibody titers. This does not necessarily indicate vaccine failure or an inadequate immune response, since repeat vaccination of previously vaccinated horses might not result in a significant increase in antibody titers.^36^ Additionally, the protection against EHV relies not only on the circulating antibodies but also on the cellular and local immune response of the host, which was not measured in this study as it is inherently difficult to quantify, and anti‐EHV serology is therefore not necessarily an exclusive marker of protective immunity and vaccination success in horses. The horses in this study had an overall markedly stronger response to the EIV antigen than the EHV antigen. In 1 study, Thoroughbred racehorses would develop higher antibody titers against EIV when they received a concurrent vaccination against EHV on the same day, without any notable decrease in the antibody levels against equine herpes virus.^37^ A similar effect could be present in the current study. The immunological explanation for and the clinical importance of this observation remains unclear.

While anti‐EHV antibody levels remained unchanged after vaccination in all the study groups, multiple doses of dexamethasone appeared to attenuate the antibody response to the EIV antigen in this study. This is in contrast to previous work in human or equine medicine, that did not find a persistently reduced antibody response to influenza virus vaccines after different dosing regimens of dexamethasone.^4^, ^5^, ^6^, ^7^ The fact that horses developed increased antibody titers against EIV even if they received a single dose of dexamethasone, might suggest a negligible immunosuppressive effect of dexamethasone on the immune response that was not measurable by routine commercial serology. However, a few reports in human and veterinary medicine suggest that a single dose of dexamethasone could have a stimulatory effect on antibody production under certain circumstances. Spickett and colleagues report increased serum antibody levels in a human patient with splenomegaly who received oral dexamethasone.^38^ Mice with latent herpes virus infection that received systemic dexamethasone or acetylcholine developed increased antibody titers compared to controls, which was attributed to reactivation of the viral infection and subsequent immune stimulation.^39^ Arnizaut and colleagues note a secondary immune response and increased serum antibody titers in cattle fish that had been experimentally infected with Ictalurid herpesvirus,^1^ when the fish received a single intramuscular dose of dexamethasone 90 days after the initial exposure to the virus.^40^

If there was a stimulatory effect of a single dose of dexamethasone on the EIV antibody production in the study horses, it was not observed among the EHV antibody levels. Exposure of the study horses to EIV during the study period could have potentially resulted in stimulation of the immune system and explained the increased antibody production against EIV but not EHV. However, this was considered unlikely since none of the horses left the premises and new horses were not introduced to any of the groups. The differences in antibody response to the bivalent vaccine could also suggest that dexamethasone might act selectively on different antigen‐specific serum immunoglobulins. Ovariectomized rats have higher postvaccine serum IgA levels but not serum IgG levels after the administration of systemic dexamethasone.^41^ Whether similar effects also occur after the vaccination of horses with a multivalent vaccine remains to be investigated.

Although the observations of this study might be specific to the available horse population and the vaccine utilized, they suggest that the effects of dexamethasone on the equine immune system are complex and incompletely understood. Different dosage regimens of dexamethasone or other corticosteroids might have different effects on the antibody response in horses and have not been sufficiently investigated. Additionally, this study has focused on the humoral immune response and therefore the effect of dexamethasone on the quality of the cell mediated immune response and the T‐cell activation in response to the EIV/EHV‐1 vaccine was not investigated. Similarly, none of the study horses were immunologically naïve before the booster vaccination, and it has been established that the secondary immune response following repeat exposure to a certain antigen will result in a more pronounced immune response mediated by the already present memory cells.^42^ Immunologically naïve horses could respond differently to corticosteroids given at the time of vaccinations.

Immune challenges following vaccination were not performed in this study but could help determine if dexamethasone affects the subsequent quality of protection of vaccinated horses against EIV or EHV‐1 infections in the absence of a significant decrease in antibody titers.

## CONFLICT OF INTEREST DECLARATION

Authors declare no conflict of interest.

## OFF‐LABEL ANTIMICROBIAL DECLARATION

Authors declare no off‐label use of antimicrobials.

## INSTITUTIONAL ANIMAL CARE AND USE COMMITTEE (IACUC) OR OTHER APPROVAL DECLARATION

Approved from the University of California, Davis IACUC, approval Date October 06, 2022, protocol number 23072, assurance Number D16‐00272.

## HUMAN ETHICS APPROVAL DECLARATION

Authors declare human ethics approval was not needed for this study.

## Supporting information


**Supplementary Information 1.** Demographics of the study horses including breed, sex, and age in years for each group. Group 1 represents the sentinel group, group 2 received the intramuscular Prestige 2 EHV‐1/4 and EIV vaccine, group 3 received the vaccine and one dose of intravenous dexamethasone (20 mg per horse) at the same time, group 4 received the vaccine and three consecutive doses of intravenous dexamethasone. The total number of horses per group, median age per group, and breed and sex distribution are summarized.

**Supplementary Table 1.** EHV‐1 serology. Total IGG and serum IG 4/7 results are expressed as median fluorescence intensity (MFI). Each row represents antibody titers for an individual horse pre‐ and postvaccination against EHV‐1. Horses are grouped according to their study group 1‐4. Study group 1 did not receive any vaccine or medication, study group 2 received only the vaccine, study group 3 received the vaccine and single dose of dexamethasone at the time of vaccination, study group 4 received the vaccine and 3 daily doses of dexamethasone.

**Supplementary Table 2.** Equine influenza serology. Prevaccine and postvaccine antibody titers against KY/14 are shown for the individual horses. The study groups are noted on the right column. Group 1 did not receive a vaccination or medications, group 2 received the vaccination only, group 3 received the vaccination and a single dose of dexamethasone at the time of vaccination, group 4 received the vaccination and 3 daily doses of dexamethasone.

## References

[1] Dauvillier J , Felippe MJ , Lunn DP , et al. Effect of long‐term fluticasone treatment on immune function in horses with heaves. J Vet Intern Med. 2011;25(3):549‐557. 21488960 10.1111/j.1939-1676.2011.0717.x

[2] Flaminio MJ , Borges AS , Nydam DV , et al. The effect of CpG‐ODN on antigen presenting cells of the foal. J Immune Based Ther Vaccines. 2007;25(5):1‐17. 10.1186/1476-8518-5-1 PMC1797044 17254326

[3] Slack J , Risdahl JM , Valberg S , et al. Effects of dexamethasone on development of immunoglobulin G subclass response following vaccination of horses. Am J Vet Res. 2000;61:1530‐1533. 11131594 10.2460/ajvr.2000.61.1530

[4] Kubiet MA , Gonzalez‐Rothi RJ , Cottey R , Bender BS . Serum antibody response to influenza vaccine in pulmonary patients receiving corticosteroids. Chest. 1996;110(2):367‐370. 8697835 10.1378/chest.110.2.367

[5] Hanania NA , Sockrider M , Castro M , et al. Immune response to influenza vaccination in children and adults with asthma: effect of corticosteroid therapy. J Allergy Clin Immunol. 2004;113(4):717‐724. 15100679 10.1016/j.jaci.2003.12.584

[6] de Roux A , Marx A , Burkhardt O , et al. Impact of corticosteroids on the immune response to a MF59‐adjuvanted influenza vaccine in elderly COPD‐patients. Vaccine. 2006;24(10):1537‐1542. 16288937 10.1016/j.vaccine.2005.10.007

[7] Inoue S , Shibata Y , Takabatake N , Igarashi A , Abe S , Kubota I . Influence of corticosteroid therapy on the serum antibody response to influenza vaccine in elderly patients with chronic pulmonary diseases. EXCLI J. 2013;12:760‐765. 26600737 PMC4653723

[8] Lee H , Punt JA , Miller DC , et al. Do corticosteroid injections for the treatment of pain influence the efficacy of mRNA COVID‐19 vaccines? Pain Med. 2021;22(4):994‐1000. 33605425 10.1093/pm/pnab063 PMC7928682

[9] Yang J , Ko JH , Baek JY , et al. Effects of short‐term corticosteroid use on reactogenicity and immunogenicity of the first dose of ChAdOx1 nCoV‐19 vaccine. Front Immunol. 2021;12:744206. 34630425 10.3389/fimmu.2021.744206 PMC8493039

[10] Accessed January 30, 2023. https://www.cdc.gov/vaccines/hcp/acip-recs/general-recs/immunocompetence.html

[11] Accessed August 10, 2023. https://inside.fei.org/system/files/Update_on_Equine_Herpes_Virus_Valencia_01_March_2021.pdf

[12] Webster WR . Overview of the 2007 Australian outbreak of equine influenza. Aust Vet J. 2011;89(Suppl 1):3‐4. 10.1111/j.1751-0813.2011.00721.x 21711267

[13] BBC News . [Internet] Equine Flu: What is the Cost to Horse Racing? Accessed August 5, 2023. https://www.bbc.com/news/business-47169299

[14] Termine C , Akerstrom G , Paixao G . Management of an EHV‐1 outbreak at FEI events and its international impact. Vet Rec. 2021;189(5):e905. 34505682 10.1002/vetr.905

[15] Walker‐Panse L , Rash A , Huckstep J , et al. Equine influenza virus surveillance in the United Kingdom from 2019 to 2021. Equine Vet J. 2021;53:78‐79.32348605

[16] Valleyvet.com . [Internet] Respiratory: Fluvac Innovator EHV‐4/1 (Rhino + Flu) Equine Vaccine . Accessed 2023 August 10. https://www.valleyvet.com

[17] Mora Pereira M , Groover E , Wooldridge A , Caldwell F . Review of glucocorticoid therapy in horses. Part 2: clinical use of systemic glucocorticoids in horses. Equine Vet Educ. 2018;30:213‐224.

[18] Leclere M . Corticosteroids and immune suppressive therapies in horses. Vet Clin North Am Equine Pract. 2017;33(1):17‐27. 28325178 10.1016/j.cveq.2016.11.008

[19] Kiemle J , Hindenberg S , Bauer N , Roecken M . Comparison of a point‐of‐care serum amyloid a analyzer frequently used in equine practice with 2 turbidimetric immunoassays used in human and veterinary medicine. J Vet Diagn Invest. 2022;34(1):42‐53. doi:10.1177/10406387211056029 34763564 PMC8688985

[20] Chambers TM , Reedy SE . Equine influenza serological methods. In: Spackman E , ed. Animal Influenza Virus. Methods in Molecular Biology. Vol 2123. New York, NY: Humana; 2020. doi:10.1007/978-1-0716-0346-8_31 32170706

[21] Wimer CL , Schnabel C , Perkins G , et al. The deletion of the ORF1 and glycoprotein 2 genes reduce virulence of the neurogenic EHV‐1 strain Ab4 without compromising host immune induction in horses. PLoS One. 2018;13(11):e02066. 10.1371/journal.pone.0206679 PMC6237298 30440016

[22] Woah.org . [Internet] Listed Disease: Equine Influenza . Accessed March 3, 2023. https://www.woah.org/fileadmin/Home/eng/Health_standards/tahm/3.06.07_EQ_INF.pdf

[23] Skipper L , Pusterla N . Correlation between serum amyloid a and antibody response to West Nile virus vaccine antigen in healthy horses. J Eq Vet Sci. 2012;106:10375. 10.1016/j.jevs.2021.103755 34670707

[24] Andersen SA , Petersen HH , Ersboll AK , et al. Vaccination elicits a prominent acute phase response in horses. Vet J. 2012;191(2):199‐202. 21371917 10.1016/j.tvjl.2011.01.019

[25] Duran MC , Dumrat CAC , Bartmann CP , et al. Serum amyloid A (SAA) concentration after vaccination in horses and mules. J Equine Vet. 2020;92:103165. 10.1016/j.jevs.2020.103165 32797788

[26] Soma LR , Uboh CE , Liu Y , et al. Pharmacokinetics of dexamethasone following intra‐articular, intravenous, intramuscular, and oral administration in horses and its effects on endogenous hydrocortisone. J Vet Pharmacol Ther. 2012;36:181‐191. 22632064 10.1111/j.1365-2885.2012.01412.x

[27] Knych HK , Weiner D , Arthur RM , Baden R , McKemie DS , Kass PH . Serum concentrations, pharmacokinetic/pharmacodynamic modeling, and effects of dexamethasone on inflammatory mediators following intravenous and oral administration to exercised horses. Drug Test Anal. 2020;12:1087‐1101. 32436346 10.1002/dta.2862

[28] Meeusen EN , Walker J , Petters A , et al. Current status of veterinary vaccines. Clin Microbiol Rev. 2007;20(3):489‐510. 17630337 10.1128/CMR.00005-07 PMC1932753

[29] Abbas AK , Lichtman AH , Pillai S . Immunity to microbes. In: Abbas AK , Lichtman AH , eds. Pillai S: Cellular and Molecular Immunology. Philadelphia, PA: Saunders; 2007:351‐373.

[30] Abbas AK , Lichtman AH , Pillai S . Innate immunity. In: Abbas AK , Lichtman AH , Pillai S , eds. Cellular and Molecular Immunology. Philadelphia, PA: Saunders; 2007:19‐46.

[31] Ellis RW . Technologies for making new vaccines. In: Plotkin S , Orenstein W , Offit P , eds. Vaccines. Philadelphia, PA: Saunders; 2008:1335‐1355.

[32] Awate S , Babiuk LA , Mutwiri G . Mechanisms of action of adjuvants. Front Immunol. 2013;4:114. 23720661 10.3389/fimmu.2013.00114 PMC3655441

[33] Felippe MJB . The immune system. In: Felippe MJB , ed. Equine Clinical Immunology. 1st ed. Ames, IA: John Wiley and Sons; 2015:1‐10.

[34] CDC.com . [Internet] Altered Immunocompetence: General Best Practice Guidelines for Immunization . Accessed June 1, 2023. www.cdc.gov/vaccines

[35] Barden A , Phillips M , Hill LM , et al. Antiemetic doses of dexamethasone and their effects on immune cell populations and plasma mediators of inflammation resolution in healthy volunteers. Prostaglandins Leukot Essent Fatty Acids. 2018;139:31‐39. 30471772 10.1016/j.plefa.2018.11.004

[36] Wagner B , Goodman LB , Babasyan S , et al. Antibody and cellular immune responses of naïve mares to repeated vaccination with an inactivated equine herpesvirus vaccine. Vaccine. 2015;33(42):5588‐5597. 26384446 10.1016/j.vaccine.2015.09.009

[37] Gildea S , Sanchez Higgins MJ , Johnson G , Walsh C , Cullinane A . Concurrent vaccination against equine influenza and equine herpesvirus—a practical approach. Influenza Other Respi Viruses. 2016;10(5):433‐437. 10.1111/irv.12396 PMC4947937 27169603

[38] Spickett GP , Zhang JG , Green T , Shrimankar J . Granulomatous disease in common variable immunodeficiency: effect on immunoglobulin replacement therapy and response to steroids and splenectomy. J Clin Pathol. 1996;49:431‐434. 8707966 10.1136/jcp.49.5.431 PMC500491

[39] Tanaka S , Mannen K . Activation of latent pseudorabies virus infection in mice treated with acetylcholine. Exp Anim. 2002;51:407‐409. 12221936 10.1538/expanim.51.407

[40] Arnizaut AB , Hanson LA . Antibody response of channel catfish after channel catfish virus infection and following dexamethasone treatment. Dis Aquat Organ. 2011;95:189‐201. 21932530 10.3354/dao02348

[41] Wira CR , Sandoe CP , Steele MG . Glucocorticoid regulation of the humoral immune system. I. In vivo effects of dexamethasone on IgA and IgG in serum and at mucosal surfaces. J Immunol. 1990;144(1):142‐146. 2295788

[42] Ahmed SA , Schurig GG , Luo XM . The specific immune response: acquired immunity. In: Klein BG , ed. Cunningham's Textbook of Veterinary Physiology. 6th ed. St Louis, MO: Elsevier; 2020:623‐624.

