# Supplementary material for: Effect of dexamethasone on antibody response of horses to vaccination with a combined equine influenza virus and equine herpesvirus‐1 vaccine
Source: J Vet Intern Med. 2023 Dec 23;38(1):424–30. doi: 10.1111/jvim.16978 (PMC10800231; doi:10.1111/jvim.16978)
Supplement: Supplementary file 1 — Supplementary Information 1. Demographics of the study horses including breed, sex, and age in years for each group. Group 1 represents the sentinel group, group 2 received the intramuscular Prestige 2 EHV‐1/4 and EIV vaccine, group 3 received the vaccine and one dose of intravenous dexamethasone (20 mg per horse) at the same time, group 4 received the vaccine and three consecutive doses of intravenous dexamethasone. The total number of horses per group, median age per group, and breed and sex distribution are summarized. [file JVIM-38-424-s003.pdf]

Group 1 (no vaccine, no dexamethasone)	Group 2 (vaccine only)	Group 3 (vaccine and one dose dexamethasone)	Group 4 (vaccine and 3 doses dexamethasone)
Quarterhorse, gelding, 17 year old<br>Warmblood, gelding, 20 year old<br>Quarterhorse, gelding, 14 year old<br>Thoroughbred, gelding, 10 year old<br>Pura Raza Espanola, gelding, 14 year old<br>Thoroughbred, gelding, 7 year old<br>Thoroughbred, mare, 11 year old<br>Quarterhorse, mare, 15 year old<br>Thoroughbred, mare, 6 year old<br>Quarterhorse, mare, 19 year old<br>Appaloosa, mare, 14 year old<br>Standardbred, mare, 13 year old<br>Warmblood, mare, 12 year old	Percheron, gelding, 19 year old<br>Quarterhorse, gelding, 18 year old<br>Quarterhorse, gelding, 14 year old<br>Bavarian Warmblood, gelding, 10 year old<br>Oldenburg, gelding, 11 year old<br>Thoroughbred, gelding, 7 year old<br>Thoroughbred, mare, 15 year old<br>Quarterhorse, mare, 14 year old<br>Quarterhorse, mare, 8 year old<br>Standardbred, mare, 18 year old<br>Irish Sports Horse, mare, 15 year old<br>Trakehner, mare, 14 year old<br>Warmblood, mare, 16 year old<br>Quarterhorse, mare, 13 year old	Quarterhorse, gelding, 6 year old<br>Arabian, gelding, 14 year old<br>Thoroughbred, gelding, 19 year old<br>German Warmblood, gelding, 10 year old<br>Thoroughbred, gelding, 12 year old<br>Thoroughbred, gelding, 14 year old<br>Thoroughbred, gelding, 14 year old<br>Quarterhorse, mare, 8 year old<br>Warmblood, mare, 9 year old<br>Thoroughbred, mare, 13 year old<br>Warmblood, mare, 11 year old<br>Thoroughbred, mare, 18 year old<br>Warmblood, mare, 16 year old<br>Thoroughbred, mare, 15 year old	Dutch Warmblood, gelding, 6 year old<br>Quarterhorse, gelding, 16 year old<br>Thoroughbred, gelding, 15 year old<br>Thoroughbred, gelding, 12 year old<br>Thoroughbred, gelding, 9 year old<br>Paint horse, gelding, 12 year old<br>Thoroughbred, gelding, 14 year old<br>Oldenburg, mare, 8 year old<br>Standardbred, mare, 9 year old<br>Thoroughbred, mare, 14 year old<br>Thoroughbred, mare, 11 year old<br>Quarterhorse, mare, 21 year old<br>Standardbred, mare, 17 year old<br>Thoroughbred, mare, 15 year old
Total: 13 horses<br>Sex: 6 gelding, 7 mares<br>median age: 14 years (range 6-20 years)<br>Breeds:<br>4 Thoroughbreds, 4 Quarterhorses, 5 others	Total: 14 horse<br>Sex: 6 geldings, 8 mares<br>median age: 14 years (range 7-19 years)<br>Breeds:<br>2 Thoroughbreds, 5 Quarterhorses, 8 others	Total: 14 horses<br>Sex: 7 geldings, 7 mares<br>median age: 14 years (range 6-19 years)<br>Breeds:<br>7 Thoroughbreds, 2 Quarterhorses, 5 others	Total: 14 horses<br>Sex: 7 geldings, 7 mares<br>median age: 14 years (range 6-21 years)<br>Breeds:<br>6 Thoroughbreds, 2 Quarterhorses, 6 others

Supplementary Information 1: Demographics of the study horses including breed, sex and age in years for each group. Group 1 represents the sentinel group, group 2 received the intramuscular Prestige 2 EHV-1/4 and EIV vaccine, group 3 received the vaccine and one dose of intravenous dexamethasone (20 mg per horse) at the same time, group 4 received the vaccine and three consecutive doses of intravenous dexamethasone. The total number of horses per group, median age per group, and breed and sex distribution are summarized.
