# Supplementary material for: Effect of dexamethasone on antibody response of horses to vaccination with a combined equine influenza virus and equine herpesvirus‐1 vaccine
Source: J Vet Intern Med. 2023 Dec 23;38(1):424–30. doi: 10.1111/jvim.16978 (PMC10800231; doi:10.1111/jvim.16978)
Supplement: Supplementary file 2 — Supplementary Table 1. EHV‐1 serology. Total IGG and serum IG 4/7 results are expressed as median fluorescence intensity (MFI). Each row represents antibody titers for an individual horse pre‐ and postvaccination against EHV‐1. Horses are grouped according to their study group 1‐4. Study group 1 did not receive any vaccine or medication, study group 2 received only the vaccine, study group 3 received the vaccine and single dose of dexamethasone at the time of vaccination, study group 4 received the vaccine and 3 daily doses of dexamethasone. [file JVIM-38-424-s001.pdf]

Total IGG pre-vaccination	Total IGG post-vaccination	Serum IG 4/7 pre vaccination	Serum IG 4/7 post vaccination	Study group
2934	2890	87	86	1
3651	3245	207	199	1
2703	2258	157	42	1
8695	8659	3278	3029	1
4256	4581	571	585	1
6332	6576	1065	1504	1
9604	10785	4635	3953	1
3234	3114	102	64	1
3061	2975	123	128	1
7149	6837	3262	2322	1
8512	8619	3603	5181	1
2126	71	2095	77	1
9280	8326	4899	3973	1
3167	3167	196	139	2
7057	7247	1690	1910	2
5682	5554	397	392	2
6380	9594	1453	5399	2
8259	7923	1281	2232	2
2051	2665	20	49	2
7314	8045	2162	3203	2
1933	2754	68	84	2
13402	12027	10583	10621	2
7000	8052	1314	2583	2
3123	5371	180	1537	2
1735	2114	37	42	2
6476	6585	916	826	2
3617	3833	51	81	2
5507	5499	1083	1139	3
7252	6759	1476	1260	3
9733	9403	3634	4903	3
7691	8929	2879	4374	3
10563	10196	5293	4884	3
6102	6662	939	1650	3
5972	8182	1029	2063	3
4517	7987	540	2990	3
4945	4833	660	905	3
5697	6563	1047	763	3
7402	9565	2309	8604	3
4664	5257	406	435	3
8322	9173	3444	4663	3

4452	5086	126	241	3
6544	5976	1158	910	4
2591	2389	62	66	4
5402	5501	870	579	4
3496	3678	218	241	4
3279	3731	143	180	4
4043	4485	338	407	4
6426	5073	977	759	4
8897	8074	3316	4095	4
5886	5476	822	608	4
6564	6804	1702	2196	4
10618	11654	5159	7267	4
14019	13772	9186	10474	4
6499	6408	1886	1935	4
4693	5431	635	670	4

Supplementary Table 1. EHV-1 serology. Total IGG and serum IG 4/7 results are expressed as median fluorescence intensity (MFI). Each row represents antibody titers for an individual horse pre- and post vaccination against EHV-1. Horses are grouped according to their study group 1 - 4. Study group 1 did not receive any vaccine or medication, study group 2 received only the vaccine, study group 3 received the vaccine and single dose of dexamethasone at the time of vaccination, study group 4 received the vaccine and 3 daily doses of dexamethasone.
