# Supplementary material for: Effect of dexamethasone on antibody response of horses to vaccination with a combined equine influenza virus and equine herpesvirus‐1 vaccine
Source: J Vet Intern Med. 2023 Dec 23;38(1):424–30. doi: 10.1111/jvim.16978 (PMC10800231; doi:10.1111/jvim.16978)
Supplement: Supplementary file 3 — Supplementary Table 2. Equine influenza serology. Prevaccine and postvaccine antibody titers against KY/14 are shown for the individual horses. The study groups are noted on the right column. Group 1 did not receive a vaccination or medications, group 2 received the vaccination only, group 3 received the vaccination and a single dose of dexamethasone at the time of vaccination, group 4 received the vaccination and 3 daily doses of dexamethasone. [file JVIM-38-424-s002.pdf]

Horse	Date	**KY/14**	**KY/14**	**Study group**
**1**	**7-Nov**	32	32	2
	**7-Dec**	32	64	
**2**	**7-Nov**	64	128	2
	**7-Dec**	128	256	
**3**	**7-Nov**	128	256	3
	**7-Dec**	128	256	
**4**	**7-Nov**	256	256	4
	**7-Dec**	256	256	
**5**	**7-Nov**	128	256	3
	**7-Dec**	128	256	
**6**	**7-Nov**	128	256	2
	**7-Dec**	128	256	
**7**	**7-Nov**	64	64	2
	**7-Dec**	128	128	
**8**	**7-Nov**	512	256	1
	**7-Dec**	512	256	
**9**	**7-Nov**	512	256	1
	**7-Dec**	128	128	
**10**	**7-Nov**	32	32	1
	**7-Dec**	32	32	
**11**	**7-Nov**	64	64	1
	**7-Dec**	64	64	

**12**	**7-Nov**	1024	1024	4
	**7-Dec**	1024	1024	
**13**	**7-Nov**	128	128	4
	**7-Dec**	128	256	
**14**	**7-Nov**	256	128	4
	**7-Dec**	256	256	
**15**	**7-Nov**	32	32	4
	**7-Dec**	64	128	
**16**	**7-Nov**	64	64	4
	**7-Dec**	128	128	
**17**	**7-Nov**	64	32	3
	**7-Dec**	128	128	
**18**	**7-Nov**	64	64	3
	**7-Dec**	128	256	
**19**	**7-Nov**	128	128	1
	**7-Dec**	256	512	
**20**	**7-Nov**	256	512	4
	**7-Dec**	256	256	
**21**	**7-Nov**	64	64	3
	**7-Dec**	256	256	
**22**	**7-Nov**	64	64	1
	**7-Dec**	256	256	
**23**	**7-Nov**	16	32	2

	**7-Dec**	256	128	
**24**	**7-Nov**	1024	1024	2
	**7-Dec**	1024	1024	
**25**	**7-Nov**	128	64	2
	**7-Dec**	64	64	
**26**	**7-Nov**	256	256	2
	**7-Dec**	256	512	
**27**	**7-Nov**	128	128	3
	**7-Dec**	256	256	
**28**	**7-Nov**	64	64	4
	**7-Dec**	128	128	
**29**	**7-Nov**	128	64	4
	**7-Dec**	128	128	
**30**	**7-Nov**	128	256	4
	**7-Dec**	256	256	
**31**	**7-Nov**	32	32	1
	**7-Dec**	32	32	
**32**	**7-Nov**	256	256	2
	**7-Dec**	512	512	
**33**	**7-Nov**	256	256	1
	**7-Dec**	256	256	
**34**	**7-Nov**	128	256	1
	**7-Dec**	128	256	

**35**	**7-Nov**	64	64	2
	**7-Dec**	128	128	
**36**	**7-Nov**	64	64	2
	**7-Dec**	256	256	
**37**	**7-Nov**	256	256	3
	**7-Dec**	512	512	
**38**	**7-Nov**	32	64	3
	**7-Dec**	128	128	
**39**	**7-Nov**	64	64	3
	**7-Dec**	64	128	
**40**	**7-Nov**	64	64	3
	**7-Dec**	128	256	
**41**	**7-Nov**	128	128	4
	**7-Dec**	256	256	
**42**	**7-Nov**	64	64	4
	**7-Dec**	128	256	
**43**	**7-Nov**	64	64	1
	**7-Dec**	128	64	
**44**	**7-Nov**	64	64	1
	**7-Dec**	128	64	
**45**	**7-Nov**	32	16	2
	**7-Dec**	64	32	
**46**	**7-Nov**	32	32	3

	**7-Dec**	128	128	
**47**	**7-Nov**	128	64	3
	**7-Dec**	128	64	
**48**	**7-Nov**	<8	1024	3
	**7-Dec**	32	32	
**49**	**7-Nov**	32	32	4
	**7-Dec**	64	64	
**50**	**7-Nov**	64	64	4
	**7-Dec**	128	128	
**51**	**7-Nov**	64	64	1
	**7-Dec**	64	64	
**52**	**7-Nov**	64	64	1
	**7-Dec**	32	32	
**53**	**7-Nov**	128	64	2
	**7-Dec**	128	256	
**54**	**7-Nov**	32	64	3
	**7-Dec**	128	128	
**55**	**7-Nov**	128	128	2
	**7-Dec**	128	128	
controls				
**Negative**		<8	<8	
**KY/14 AS**		1024	1024	

Supplementary Table 2. Equine influenza serology. Pre-vaccine and post-vaccine antibody titers against KY/14 are shown for the individual horses. The study groups are noted on the right column. Group 1 did

not receive a vaccination or medications, group 2 received the vaccination only, group 3 received the vaccination and a single dose of dexamethasone at the time of vaccination, group 4 received the vaccination and 3 daily doses of dexamethasone.
